# Colonic insufflation with carbon monoxide gas inhibits the development of intestinal inflammation in rats

**DOI:** 10.1186/2045-9912-2-23

**Published:** 2012-09-03

**Authors:** Tomohisa Takagi, Yuji Naito, Kazuhiko Uchiyama, Toshimitsu Okuda, Takahiro Suzuki, Hisato Tsuboi, Katsura Mizushima, Osamu Handa, Nobuaki Yagi, Hiroshi Ichikawa, Toshikazu Yoshikawa

**Affiliations:** 1 Molecular Gastroenterology and Hepatology, Graduate School of Medical Science, Kyoto Prefectural University of Medicine, 465 Kajii-cho, Kawaramachi-Hirokoji, Kamigyo-ku, Kyoto, 602-8566, Japan

**Keywords:** Carbon monoxide (CO), Insufflation, 2,4,6-Trinitrobenzene sulfonic acid (TNBS)-induced colitis, Inflammatory bowel disease (IBD)

## Abstract

**Background:**

The pathogenesis of inflammatory bowel disease (IBD) is complex, and an effective therapeutic strategy has yet to be established. Recently, carbon monoxide (CO) has been reported to be capable of reducing inflammation by multiple mechanisms. In this study, we evaluated the role of colonic CO insufflation in acute colitis induced by trinitrobenzene sulfonic acid (TNBS) in rats.

**Methods:**

Acute colitis was induced with TNBS in male Wistar rats. Following TNBS administration, the animals were treated daily with 200 ppm of intrarectal CO gas. The distal colon was removed to evaluate various parameters of inflammation, including thiobarbituric acid (TBA)-reactive substances, tissue-associated myeloperoxidase (MPO) activity, and the expression of cytokine-induced neutrophil chemoattractant (CINC)-1 in colonic mucosa 7 days after TNBS administration.

**Results:**

The administration of TNBS induced ulceration with surrounding edematous swelling in the colon. In rats treated with CO gas, the colonic ulcer area was smaller than that of air-treated rats 7 days after TNBS administration. The wet colon weight was significantly increased in the TNBS-induced colitis group, which was markedly abrogated by colonic insufflation with CO gas. The increase of MPO activity, TBA-reactive substances, and CINC-1 expression in colonic mucosa were also significantly inhibited by colonic insufflation with CO gas.

**Conclusions:**

Colonic insufflation with CO gas significantly ameliorated TNBS-induced colitis in rats. Clinical application of CO gas to improve colonic inflammatory conditions such as IBD might be useful.

## Background

Inflammatory bowel disease (IBD) consists of chronic and relapsing inflammatory diseases of the intestines; the pathogenesis of IBD, including Crohn’s disease (CD) and ulcerative colitis (UC), is complex. Although it has been reported that genetic, immunologic, and environmental factors are involved in the initiation and perpetuation of chronic intestinal inflammation [1,2], the precise pathogenesis remains unclear. 5-aminosalicylates (5-ASA), corticosteroids, immunosuppressive agents, or anti-tumor necrosis factor (TNF)-α antibodies are typically used for the management of IBD. However, a substantial number of patients of IBD experience relapse or an incomplete response to these therapies.

The role of carbon monoxide (CO), a component of cigarette smoke, has been reported [3] to provide protection against chronic intestinal inflammation. Although CO is classified as a toxic agent that is potentially lethal and is a major pollutant in industrialized society, CO has recently emerged as a potent immunomodulatory entity, anti-inflammatory agent, and an important factor in physiological homeostasis [4-7]. The anti-inflammatory effect of CO has been reported in various disease states and experimental models, including ischemia-reperfusion injury [8,9], organ transplantation [10], hyperoxia [11], and lipopolysaccharide (LPS)-induced sepsis [12]. In these conditions, CO-mediated protection is associated with suppression of the inflammatory cytokine response. We have previously reported that CO inhalation ameliorates 2,4,6-trinitrobenzene sulfonic acid (TNBS)-induced murine colitis through TNF-α expression in CD4^+^ T cells [13]. CO-releasing molecule (CORM)-2 also suppressed colonic inflammation induced by dextran sulfate sodium (DSS) in mice [14]. In addition, CO inhalation suppressed inflammation in a genetically induced mouse colitis model, in interleukin (IL)-10-deficient mice [15], and in T cell receptor (TCR)α-deficient mice [16]. Based on these reports, CO administration might represent a potential therapeutic strategy for IBD.

In the present study, we demonstrated the beneficial effect of CO in the colonic inflammatory condition by using a TNBS-induced colitis model in rats with insufflation of CO gas into the colonic lumen.

## Methods

### Animals

Male Wistar rats weighing 180–200 g were obtained from SHIMIZU Laboratory Supplies Co. Ltd. (Kyoto, Japan). The animals were housed at 22°C in a controlled environment with 12 h of artificial light per day; they were allowed access to rat chow and water *ad libitum*. The animals were maintained and all experimental procedures were carried out in accordance with the National Institutes of Health (NIH) guidelines for the use of experimental animals. All experimental protocols were approved by the Animal Care Committee of the Kyoto Prefectural University of Medicine (Kyoto, Japan).

### TNBS-induced colitis in rats

Colitis was induced by the previously described method [17]. In brief, the rats were anesthetized with pentobarbital sodium (Kyoritsu Seiyaku Corporation, Tokyo, Japan). Following lower abdominal laparotomy, the colon was exposed. The middle portion of the colon was pinched with ring forceps (inside diameter, 8 mm), and 0.2 mL of 30% ethanol solution containing a final concentration of 0.2 M TNBS (Sigma-Aldrich Japan, Tokyo, Japan) was injected into the luminal side of the clamped portion of the colon. After 2 min, the colon was returned to the abdominal cavity and the incision was sutured. All procedures apart from TNBS injection were performed in rats in the sham-operated control group.

### Treatment protocol

Following TNBS administration, the animals were treated daily with 200 ppm of CO gas. Colonic CO gas insufflation (volume, 8 mL) was performed using a rubber catheter (outer diameter, 2 mm) via the anus under light anesthesia with diethyl ether (Wako Pure Chemicals, Osaka, Japan). The colonic insufflation with CO gas performed twice a day for 7 days after the induction of TNBS colitis in rats. All animals were randomized into groups treated with colonic CO gas insufflation or air insufflation.

The rats were sacrificed 7 days after TNBS treatment, and the distal colon was removed and opened by longitudinal incision. The wet colon weight was measured immediately thereafter. The size of the ulcer was also measured, and the ulcer index was calculated from the resultant length and width measurements (mm^2^). For histologic evaluation, formalin-fixed tissues were stained with hematoxylin and eosin and evaluated by light microscopy.

### Measurements of thiobarbituric acid (TBA)-reactive substances and myeloperoxidase (MPO) activity

As an index of lipid peroxidation, the total concentration of TBA-reactive substances was measured in the intestinal mucosa as previously described [13,18]. Briefly, the colonic mucosa was scraped off using 2 glass slides, and was then homogenized with 1.5 mL of 10 mM potassium phosphate buffer (pH 7.8) containing 30 mM KCl. The level of TBA-reactive substances in the mucosal homogenates was expressed as nmoles of malondialdehyde/mg of protein, using 1,1,3,3-tetramethoxypropane as the standard. The total protein in the tissue homogenates was measured with a Bio-Rad Protein Assay kit (Bio-Rad Laboratories, K. K., Tokyo, Japan) according to the manufacturer’s protocol.

Tissue-associated MPO activity in the intestinal mucosa was determined as an index of neutrophil accumulation as described elsewhere [13,18]. The mucosal homogenates were centrifuged at 20,000 × *g* for 15 min at 4°C to collect the insoluble cellular debris. The resultant pellet was then homogenized in an equivalent volume of 0.05 M potassium phosphate buffer (pH 5.4) containing 0.5% hexadecyltrimethylammonium bromide. The samples were centrifuged at 20,000 × *g* for 15 min at 4°C, and the supernatants were saved. MPO activity was assessed by measuring the H_2_O_2_-dependent oxidation of 3,3^′^,5,5^′^-tetramethylbenzidine. One unit of enzyme activity was defined as the amount of MPO required to cause a change in absorbance of 1.0/min at 645 nm and 25°C.

### Determination of the colonic mucosal content and mRNA expression of cytokine-induced neutrophil chemoattractant (*CINC*)*-1*

The concentration of rat CINC-1, a potent member of the IL-8 family [19,20], in the colonic mucosal homogenates was determined with the Rat GRO/CINC-1 Assay kit (Immuno-Biological Laboratories Co., Ltd., Gunnma, Japan), according to the manufacturer’s instructions.

The colonic mucosal mRNA expression of *CINC-1* (and *β-actin* as the internal control), was determined by real-time PCR according to the protocol used in our previous study [13,14]. Tissue samples for mRNA isolation were removed from colonic mucosa. Total RNA was isolated by the acid guanidinium-phenol-chloroform method with Isogen (Nippon Gene Co. Ltd., Tokyo, Japan). The isolated RNA was stored at −70°C until use in real-time PCR. For real-time PCR, 1 μg of extracted RNA was reverse-transcribed into first-strand complementary DNA (cDNA) using the High Capacity cDNA Reverse Transcription Kit (Applied Biosystems, Foster City, CA, USA). Real-time PCR for *CINC-1* and *β-actin* performed with a 7300 Real-Time PCR system (Applied Biosystems) using the DNA-binding dye SYBR® Green for the detection of PCR products. The primers had the following sequences: *CINC-1* sense, 5^′^-CCATTAAGTGTCAACCACTGTGCTA-3^′^; *CINC-1* antisense, 5^′^-CACATTTCCTCACCCTAACACAAA-3^′^; *β-actin* sense, 5^′^-GAGC AAACATCCCCCAAAGTT-3^′^; and; *β-actin* antisense, 5^′^-GCCGTGGATACTTGGAGTGACT-3^′^. Relative quantification of gene expression from the real-time PCR data was calculated relative to *β-actin* expression.

### Statistical analysis

The results are presented as the mean ± standard error of the mean (SEM). Overall differences between the groups were determined by one-way analysis of variance (ANOVA). For cases in which the one-way ANOVA was significant, differences between individual groups were analyzed by Bonferroni’s multiple comparisons test. Differences in which *P* < 0.05 were considered significant. All analyses were performed using the GraphPad Prism 4 program (GraphPad Software Inc., San Diego, CA, USA) for Macintosh.

## Results

### Effect of colonic insufflation with CO gas on TNBS-induced colitis

**Figure 1:**
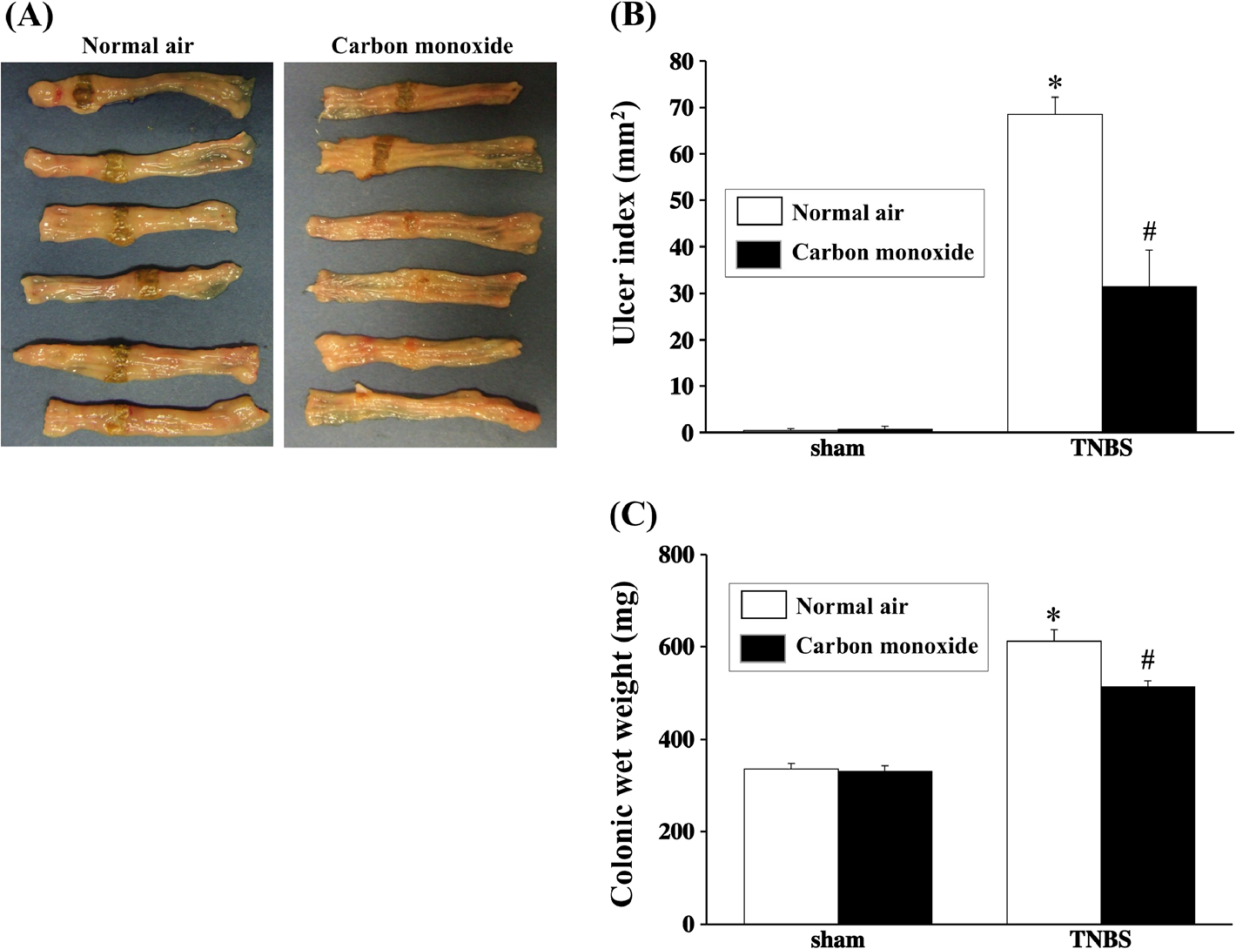
**Effects of CO insufflation into the colonic lumen on macroscopic findings, mucosal damage score, and wet colon weight after trinitrobenzene sulfonic acid (TNBS)-induced injury.** (**A**) Severe colitis was induced, including hyperemia, edema, thickening, ulceration, and necrosis, in TNBS-treated rats (normal air group). These changes were reduced in rats treated by CO insufflation into the colonic lumen (CO group). (**B**) The ulcer index was evaluated. *P < 0.01 compared to sham-operated rats. ^#^*P* < 0.05 compared to rats with TNBS-induced colitis receiving normal air insufflation. (**C**) The wet colon weight was measured. Data represent the mean ± SEM of 7 rats. *P < 0.05 relative to sham-operated rats. ^#^*P* < 0.05 relative to rats with TNBS-induced colitis receiving normal air insufflation.

After 7 days of treatment with TNBS, macroscopic findings in the colon demonstrated severe colonic ulceration, with a marked difference in margin relative to the normal mucosa in addition to surrounding edematous swelling. In rats treated with colonic CO gas insufflation, the area of the colonic ulcer was smaller than that of air-treated rats (Figure 1A). More precisely, while the ulcer index was 68.5 ± 3.7 mm^2^ in control rats, the ulcer index in rats treated with colonically insufflated CO gas was 31.3 ± 7.9 mm^2^ (Figure 1B). Furthermore, the wet colon weight was significantly increased in the TNBS colitis group (air-treated rats). This increase was significantly ameliorated by treatment with colonically insufflated CO gas (Figure 1C).

**Figure 2:**
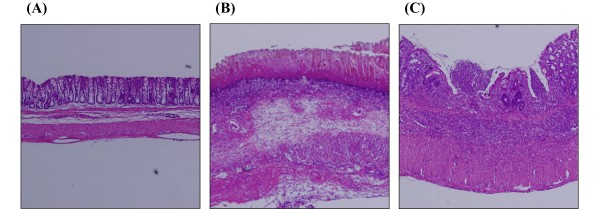
**Effects of colonic insufflation with CO gas on histological findings in the colon 7 days after trinitrobenzene sulfonic acid (TNBS)-induced injury.** Histological appearance of colonic tissue in sham-operated rats (**A**), rats with TNBS-induced colitis (**B**), and rats with TNBS-induced colitis treated with colonic insufflation of CO gas (**C**). Histological examination revealed that TNBS administration induced marked thickening of the colonic wall and colonic ulceration, which was associated with transmural infiltration of inflammatory cells. In contrast, both wall thickening and colonic ulceration were reduced in rats treated with colonic insufflation of CO gas. Hematoxylin and eosin (H&E) staining is shown (40 × magnification).

The effects of treatment with the colonically insufflated CO gas were also confirmed by histological examination. Figure 2 shows the representative histological features of a normal colon in sham-operated rats (A), those of TNBS-induced colitis group (treated with air, B) and those of the CO gas-treated group (C). The administration of TNBS induced a marked thickening of the colonic wall and large colonic ulceration with transmural infiltration of numerous inflammatory cells (Figure 2B), as compared to the normal colon (Figure 2A). However, in rats treated with CO gas, an inhibition of both mural wall thickening and colonic ulceration was observed (Figure 2C).

### Effect of colonic insufflation with CO gas on TBA-reactive substances and MPO activity

**Figure 3:**
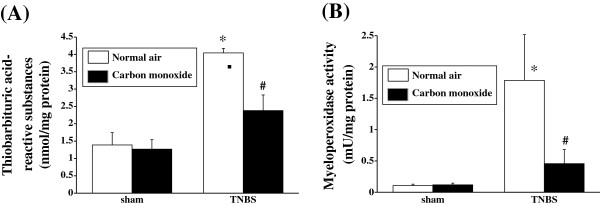
**Effects of CO insufflation into the colonic lumen on thiobarbituric acid (TBA)-reactive substances and tissue-associated myeloperoxidase (MPO) activity.** The level of TBA-reactive substances (**A**) and MPO activity (**B**) were significantly inhibited in rats treated with CO gas insufflation. Data represent the mean ± SEM of 7 rats. *P < 0.05 relative to sham-operated rats. ^#^*P* < 0.05 relative to rats with TNBS-induced colitis receiving normal air insufflation.

The extent of lipid peroxidation was determined by measuring the TBA-reactive substances present in the colonic mucosa. In the sham-operated group, there were no differences in the levels of intestinal TBA-reactive substances between CO gas- and normal air-treated rats. However, the TNBS-induced colitis caused a significant increase in TBA-reactive substances compared to that of the sham-operated rats. The increase in TBA-reactive substances in the colonic mucosa was significantly inhibited by colonic insufflation with CO gas (Figure 3A).

Neutrophil accumulation was also evaluated by the measurement of tissue-associated MPO activity in colonic mucosal homogenates. In the sham-operated animals, there were no differences in the MPO activities between CO gas- and normal air-treated rats. In contrast, MPO activity in the colonic mucosa was markedly increased in animals with TNBS-induced colitis relative to that of the sham-operated group. The MPO activity in the colonic mucosa after the induction of colitis with TNBS was significantly inhibited by treatment with colonic CO gas insufflation (Figure 3B).

### Effect of colonic insufflation with CO gas on CINC-1 protein and mRNA expression in the colonic mucosa

**Figure 4:**
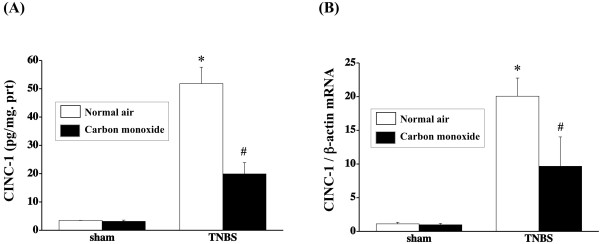
**Effects of CO insufflation into the colonic lumen on CINC-1 expression.** The CINC-1 protein expression level (**A**) and mRNA level (**B**) were significantly inhibited in rats treated with CO gas insufflation. Data represent the mean ± SEM of 7 rats. *P < 0.01 relative to sham-operated rats. ^#^*P* < 0.05 relative to rats with TNBS-induced colitis receiving normal air insufflation.

To further analyze the effects of the colonic insufflation with CO gas on neutrophil accumulation in the colonic mucosa, we assessed the colonic mucosal CINC-1 protein level using an enzyme-linked immunosorbent assay (ELISA) and *CINC-1* mRNA expression using real time-PCR. The colonic CINC-1 protein level increased significantly after the induction of colitis with TNBS. The increase in CINC-1 in the colonic mucosa was significantly inhibited by colonic insufflation with CO gas (Figure 4A). RNA extracts obtained from the colon were subjected to real-time PCR to measure *CINC-1* gene expression. As shown in Figure 4B, subtle expression of the *CINC-1* gene was revealed in sham-operated rats, while *CINC-1* transcription was enhanced in the TNBS-treated rats. Treatment with CO gas suppressed *CINC-1* mRNA expression in the inflamed colonic tissue (Figure 4B).

## Discussion

In the present study, we demonstrated that insufflation of CO gas into the colonic lumen decreased colonic mucosal damage and inflammation induced by TNBS. To our knowledge, this is the first report demonstrating the anti-inflammatory effect of insufflation of CO gas into the colonic lumen. The pathogenesis of IBD such as CD and UC are complicated and remain unclear. Concurrently, although new treatment modalities such as immunosuppressants and anti-TNF-α antibodies have been proposed for the treatment of IBD, the progress of disease remains poorly controlled in some patients. We suggest the possibility of the clinical application of CO gas to control IBD.

Cigarette smoking has been reported to have a protective effect against the development of UC [21,22]. Although the detailed mechanisms remain unclear, CO—a component of cigarette smoke—has been reported to abrogate colonic inflammation. The ability of CO to inhibit colonic mucosal inflammation has been reported in an experimental colitis model. IL-10-deficient (IL-10^−/−^) mice develop chronic colitis, which is mediated by T-helper (Th)-1 cytokines. CO exposure at a concentration of 250 ppm for 7 days ameliorated colitis in IL-10^−/−^ mice [15]. In Th-1 mediated inflammation, CO decreases the synergistic effect of interferon (IFN)- γ on LPS-induced IL-12 p40 in murine macrophages. In addition, CO exposure has been reported to alleviate chronic colitis in TCRα^−/−^ mice in which the disease is mediated by Th-2 cytokines [16]. CO exposure at a concentration of 250 ppm for 7 days decreased the colitis score and inflammatory cytokine expression in the colonic mucosa. In that model, CO induced heme oxygenase (HO)-1 expression that was correlated with increased IL-10 and IL-22 expression in macrophages, suggesting that HO-1 induction by CO may be associated with anti-inflammatory mechanisms. In our previous study, TNBS-induced murine colitis was also improved by CO inhalation [13]. CO inhalation significantly decreased the macroscopic colonic damage score, amount of TBA-reactive substances, and MPO activity in the colonic mucosa. Not only the expression of TNF-α in the colonic mucosa, but also TNF-α production by CD4^+^ T cells isolated from the spleen was significantly inhibited by treatment with inhaled CO. Furthermore, CORM-2 improved colonic mucosal inflammation and damage in an experimental colitis model as well [14]. The disease activity index (DAI) score and MPO activity in the colonic mucosa were significantly decreased by treatment with CORM-2 in an acute mouse colitis model induced by DSS. The expression of inflammatory cytokines such as TNF-α and chemokines such as keratinocyte chemoattractant (KC) were decreased after CORM-2 treatment.

In the present study, we administered CO into the colonic lumen of rats, and CO insufflation significantly improved the ulcer index and decreased wet colon weight in a TNBS-induced colitis model. The level of TBA-reactive substances and MPO activity were also decreased after CO insufflation. The elevation of TBA-reactive substances is a reliable indicator of lipid peroxidation, which is closely associated with tissue damage [23]. Since oxidative stress in the colonic mucosa is closely related to neutrophil infiltration, the elevation of TBA-reactive substances is suggested to be a subsequent event of the elevation of MPO activity. These results indicate that CO insufflation decreased neutrophil infiltration and subsequent mucosal damage. In addition, we confirmed that the induction of CINC-1 in the colonic mucosa by TNBS was significantly inhibited by CO treatment. CINC-1 is a homolog of human IL-8 and plays an important role in the acute phase of inflammation [24,25]. Although further study is necessary to elucidate the detail mechanism by which CO suppresses CINC-1 expression in the colonic mucosa, it is clear that CO insufflation significantly reduced the expression of inflammatory mediator CINC-1, which might result in reduced mucosal inflammation.

More importantly, blood CO concentration was not elevated after CO insufflation in the colonic lumen in this study (data not shown), indicating that the rectal administration of CO gas might be safety and realistic route for clinical application though the inhalation of CO gas caused the high toxicity through the high concentration of CO in blood. In addition, the unchanging CO concentration in blood after the rectal administration of CO gas may indicate that CO act topically in colonic mucosa. In previous study using murine colonic epithelial cells, we demonstrated that CO inhibited the production of keratinocyte chemoattractant (KC), which has represented a closely related chemokine involved in neutrophil recruitment [26] and been regarded as a functional homologue of IL-8, through the inhibition of NF-κB activation [14]. Similar to these results, Megias et al. also described that CO inhibited IL-8 production in a human colonic epithelial cell line, Caco-2, through the inhibition of NF-κB activation [27]. In view of the results from these recent investigations, the inhibition of cytokine production might be an essential mechanism by which CO yields anti-inflammatory effects.

For the management of IBD, since mucosal healing is associated with a better outcome with decreased risk of relapse and major surgery, direct assessment of severity and mucosal healing using endoscopy is critical [28,29]. In particular, for combination therapy with infliximab and azathioprine in CD patients, endoscopy may help to identify patients who will experience the best outcome due to early intervention [30]. However, an association between colonoscopy preparation and toxic megacolon in severe UC has been suggested [31]. It has also been reported that left-sided colonic mucosal ulcerations might be induced by sodium phosphate preparation and polyethyleneglycol [32,33]. Although the precise etiology of this association is unknown, an increase in crypt cell apoptosis has been suggested to be an important mechanism of mucosal damage due to colonoscopy preparation [34]. An association between colonoscopy and exacerbation of UC symptoms has been also reported [35]. As a constant air supply during the endoscopic examination is indispensable for observing the colonic mucosa, we suggest that CO insufflation used instead of the normal air supply during endoscopic examination of IBD patients might prevent colonic mucosal damage induced by the preparation for colonoscopy.

## Conclusions

In conclusion, we demonstrated the beneficial effect of CO insufflation into the colonic lumen to decrease mucosal inflammation. Although additional research is required before CO gas can be used for clinical applications, it might be a useful new complementary therapeutic strategy for the management of IBD.

## Abbreviations

IBD: Inflammatory bowel disease; CD: Crohn’s disease; UC: Ulcerative colitis; 5-ASA: 5-Aminosalicylates; TNF: Tumor necrosis factor; CO: Carbon monoxide; LPS: Lipopolysaccharide; TNBS: 2,4,6-Trinitrobenzene sulfonic acid; CORM: CO-releasing molecule; DSS: Dextran sulfate sodium; IL: Interleukin; TCR: T cell receptor; TBA: Thiobarbituric acid; MPO: Myeloperoxidase; CINC: Cytokine-induced neutrophil chemoattractant; SEM: Standard error of the mean; ANOVA: Analysis of variance; ELISA: Enzyme-linked immunosorbent assay; IFN: Interferon; HO: Heme oxygenase; DAI: Disease activity index; KC: Keratinocyte chemoattractant.

## Competing interests

Yuji Naito received scholarship funds from Otsuka Pharmaceutical Co., Ltd. and Takeda Pharmaceutical Co., Ltd.

Nobuaki Yagi has an affiliation with a donation-funded department from AstraZeneca Co., Ltd., Eisai Co., Ltd., Otsuka Pharmaceutical Co., Ltd., MSD K.K., Dainippon Sumitomo Pharma Co., Ltd., Chugai Pharmaceutical Co., Ltd., FUJIFILM Medical Co., Ltd. and Merck Serono Co., Ltd.

The other authors declare that there are no competing interests.

## Authors’ contributions

TT, TO, TS, and HT performed rat experiments. TT and YN participated in the design of the study. KM and OH performed the gene expression analysis. NY performed the measurement of biochemical index such as MPO activity. HI performed the statistical analysis. KU helped to draft the manuscript. TY provided overall supervision. All authors read and approved the final manuscript.
